# Mechanical and vibrational behaviors of bilayer hexagonal boron nitride in different stacking modes

**DOI:** 10.1038/s41598-024-61486-5

**Published:** 2024-05-09

**Authors:** Demin Zhao, Kexin Fang, Zhilong Lian

**Affiliations:** 1 College of Pipeline and Civil Engineering, China University of Petroleum (East China), Qingdao, 266580 People’s Republic of China; 2 CNPC Engineering Technology Institute Ltd, Beijing, 102206 People’s Republic of China

**Keywords:** Bilayer hexagonal boron nitride, Five stacking mode, Tensile behavior, Natural frequency, Molecular dynamics simulation, Engineering, Nanoscience and technology

## Abstract

Hexagonal boron nitride (h-BN) is a semiconductor material with a wide band gap, which has great potential to serve as a nanoresonators in microelectronics and mass and force sensing fields. This paper investigates the mechanical properties and natural frequencies of bilayer h-BN nanosheets under five different stacking modes, which have been rarely studied, using molecular dynamics simulations. The mechanical properties, including Young’s modulus, the ultimate stress, ultimate strain, Poisson’s ratio and shear modulus, are studied for all five stacking modes. And the effects of strain rate, crystal orientation and temperature to bilayer h-BN nanosheets’ tensile properties have also been studied. Our findings suggest that bilayer h-BN nanosheets are basically an anisotropic material whose tensile properties vary substantially with stacking modes and temperature. Moreover, the natural frequencies are proposed in an explicit form based on the nonlocal theory. The differences of the fundamental natural frequencies among different stacking modes are affected by the constraint condition of bilayer h-BN sheet. The theory results match well with the simulation results. These findings establish elementary understandings of the mechanical behavior and vibration character of bilayer h-BN nanosheets under five different stacking modes, which could benefit its application in advanced nanodevices.

## Introduction

It is well known that the progress of science and technology is largely driven by new nanoscale materials^1–4^, such as graphene, diamond, MoS_2_ and boron nitride. In the past 2 decades, many nanoscale functional devices have been explored due to the excellent performance exhibited by nanostructured materials, which provides many new opportunities for various science and engineering fields. For example, diamond nanobelts exhibit ultra-high vibrational response are expected to serve as nanoscale mass sensors^5^, boron nitride nanotube inorganic composite materials with good piezoelectric properties have the potential to be used as nanogenerators^6^, square graphene flakes which can detect inert gases could be used as mass sensors to detect rare gases and other substances. All of these examples indicate that nanoscale structured materials have broad prospects for applications as sensors, detectors, and nanoelectromechanical systems (NEMS).

Boron nitride, a single-layer nanomaterial with a structure similar to graphene, has drawn a great interest recently. As the III–V binary compound of graphene, it is also a typical two-dimensional nanomaterial with four forms of boron nitride: hexagonal boron nitride (h-BN), cubic boron nitride (c-BN), wurtzite boron nitride (w-BN), and rhombohedral boron nitride (r-BN)^7^. H-BN has structural properties similar to graphene, as well as high chemical stability, high elastic modulus, low mass density, and excellent thermal properties^8,9^. These unique properties indicate that h-BN, as a promising nanomaterial^10^, has considerable potential to exhibit excellent performance in microelectronics, optoelectronics, sensing, and other fields. In addition, h-BN has many unique properties compared to graphene. For example, compared to graphene’s zero bandgap, h-BN has a very wide bandgap^11–13^, which makes it a promising resonator in wide-bandgap 2D NEMS. Studies have also found that nanomechanical resonators can easily achieve very high resonant frequencies, up to gigahertz, even in very small areas on chips^14^. Moreover, due to the piezoelectric properties of h-BN, its resonant frequency can be effectively tuned by applying an external electric field^15,16^, which indicates that h-BN can be used to design new piezoelectrically tunable 2D nanoresonators. Furthermore, h-BN has excellent properties such as higher thermal conductivity, higher radiation resistance, and larger electron saturation drift velocity, making it more suitable for extreme environments such as high temperature, high power, high voltage and strong radiation^17–20^.

So far, there have been extensive studies on the mechanical properties and vibrational characteristics of single-layer h-BN nanosheets, such as their vibrational behavior under the influence of an electric field^16^, the effects of temperature and strain rate on the tensile properties of single-layer h-BN nanosheets^9^. There have also been many studies on boron nitride nanotubes, such as the axial tensile deformation behavior^17^, torsional characteristics^21^, and axial elastic properties^22^ of boron nitride nanotubes. But it is worth noting that although the boron nitride crystal is very similar to graphene, the boron nitride crystal is a honeycomb structured atomic thin film stack, where boron(B) and nitrogen(N) atoms can occupy different positions in the lattice honeycomb structure^23^. Furthermore, although the structure of a single-layer of h-BN nanosheets is almost identical to that of graphene, under the interlayer Coulomb interaction of a bilayer h-BN nanosheet, the polar B and N atoms may have different stacking modes which resulting in five different stacking patterns^24^. However, there is a lack of comprehensive understanding of the mechanical properties and vibrational characteristics of bilayer h-BN nanosheets in different stacking modes. Therefore, based on the various properties of single-layer h-BN nanosheets and nanotubes, it is extremely necessary to further investigate the tensile and vibrational properties of bilayer h-BN nanosheets.

All the MD simulations in this paper are carried out using LAMMPS (Large-scale Atomic/Molecular Massively Parallel Simulator), an open-source package developed by Sandia National Laboratories^25^. We investigate the tensile and shear behaviors of bilayer h-BN nanosheets in five different stacking modes. Additionally, the effects of the crystal orientation and the temperature on the mechanical properties of h-BN are also taken into consideration. Furthermore, the vibrational behavior of the bilayer h-BN nanosheets in five stacking modes is studied using MD simulations. Finally, we employ nonlocal theory at the nanoscale to model the bilayer h-BN nanosheets and propose an explicit expression for the natural frequencies of bilayer h-BN nanosheets, which verify the accuracy of the MD simulations. Hoping that these research findings can provide important guidance for the practical applications of h-BN in the field of nanodevices.

## Model and method

**Figure 1:**
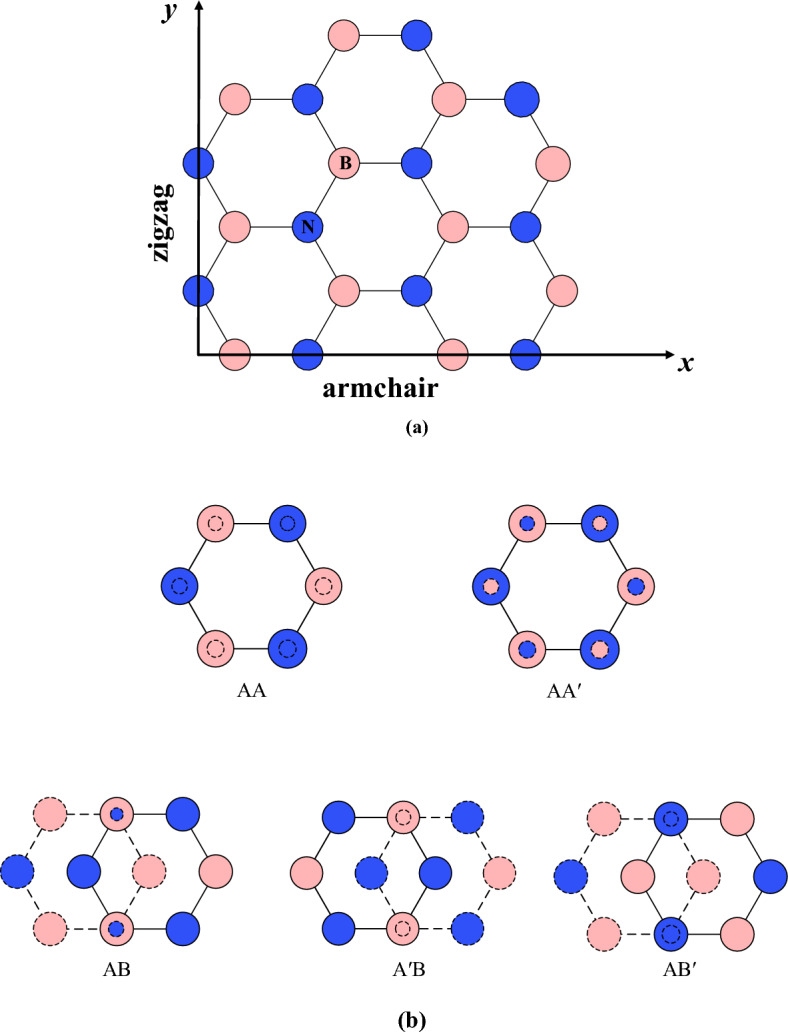
(**a**) Atomic structure of h-BN nanosheets from top view, *x* and *y* axes are chosen to lie in the armchair and zigzag directions, respectively. (**b**) The five different stacking modes of bilayer h-BN from top view.

In this study, the schematic diagrams of h-BN and the different stacking modes of bilayer h-BN nanosheets involved in the crystal are shown in Fig. 1, where the ochre and blue atoms represent B and N atoms, respectively. Moreover, the h-BN crystals have a planar honeycomb hexagonal structure, which is similar to its graphene counterparts, and have two principal directions, armchair and zigzag directions shown in Fig. 1. The armchair and zigzag directions are oriented along the *x* and *y* axes, respectively. According to the different relative locations of B and N in upper and bottom layer, the five stacking modes of bilayer h-BN nanosheets are denoted as AA, AA′, AB, A′B and AB′, respectively.

**Figure 2:**
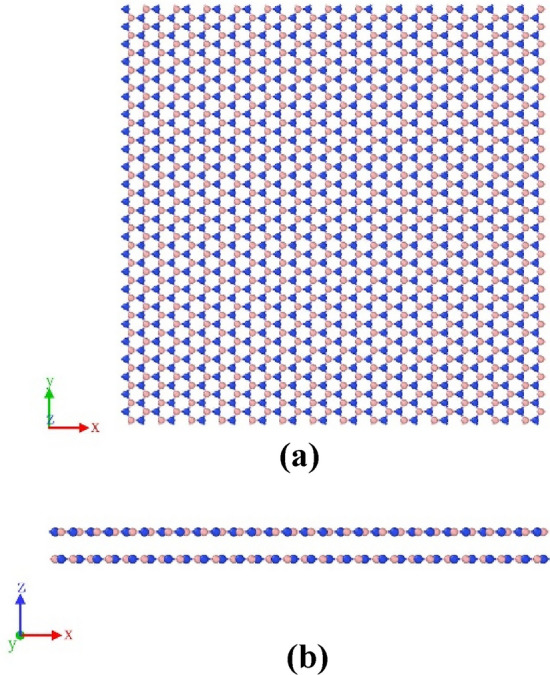
The simulation model of the h-BN AA′ nanosheets tested in this study. (**a**) top view; (**b**) front view.

Under five different stacking modes, the size of the bilayer h-BN nanosheets for the simulation model are all the same and the structures are rectangular shown in Fig. 2, containing 2688 atoms with a length of 60 Å (armchair direction) and a width of 60 Å (zigzag direction).

The potential function describes the interaction energy and forces between atoms in the system during the MD simulation, and the choice of potential function has a significant impact on the simulation results. Therefore, we need to select an appropriate potential function before conducting MD simulations. Due to the accurate description of mechanical and thermal properties between B and N atoms in boron nitride nanosystems by the Tersoff-Bernner potential^26–28^, it is employed in all simulations in this study to characterize the interaction between B and N atoms. The Tersoff–Brenner^28^ potential function used in this study is expressed as follows:

1 $$ E = \frac{1}{2}\sum\limits_{i \ne j}^{{}} {V_{ij}^{T} \left( {r_{ij} } \right) + } F_{ij} \left( {r_{ij} } \right) + V_{ij}^{B} \left( {r_{ij} } \right) $$

The first term of this potential function corresponds to the influence of bond length and bond angle, and the second term denotes the three-body angle on energy loss, while the third term is correction term which considers the interaction between B and N. Here, *E* is the energy of the system, *V*_*ij*_ is the pair energy of the pair of atoms and *r*_*ij*_ is the length of the *ij* bond and the indices *i* and *j* run over all atoms. Although the Tersoff–Brenner potential is often adopted to describe the interactions between boron and nitride atoms for the study of single-layer boron nitride nanosystems^29^, the forces between layers of the bilayer h-BN nanosheets in this study cannot be described by this potential. The forces between the interlayers of nanosheets, caused by the instantaneous dipole-induced dipole interaction, are typically described by van der Waals interactions^30–32^, which can be described by the long-range Lennard–Jones (LJ) 12–6 potential as the following form:

2 $$ E_{{{\text{LJ}}}} = 4\chi \left[ {\left( {\frac{\delta }{r}} \right)^{12} - \left( {\frac{\delta }{r}} \right)^{6} } \right] \left( {r < r_{c} } \right), $$

where *r*_*c*_ is the cutoff between two atoms, *χ* is the depth of the potential well, and *δ* is the distance at which the potential becomes zero. To model the interactions between B and N atoms between layers, we adjust the LJ parameters using follow equation^32^:

3 $$ \chi = \sqrt {\chi_{i} \chi_{j} } , $$

4 $$ \delta = \left( {\delta_{i} + \delta_{j} } \right)/2, $$

where *i* and *j* refer to B and N. The adjusted results are given in Table 1.

**Table 1:** Parameters of the LJ potential for the interactions between boron and nitrogen.

	B–B	N–N	B–N
$\chi$ (eV)	0.004116	0.006281	0.005084
$\delta$ (Å)	3.453	3.365	3.409

However, it is worth noting that while previous studies^1,29,33^ mostly used the LJ potential to describe interlayer van der Waals forces, some recent scholars have suggested that the LJ potential may not accurately represent the interlayer van der Waals interactions between two-dimensional layered materials^34–37^. In contrast, the KC potential function can more accurately describe interlayer van der Waals forces. This paper also explores the differences in results between the LJ and KC potentials, and in some simulations, opts for the KC potential, which may be closer to predicting mechanical properties.

To simulate the tensile, shear, and vibrational properties of the bilayer h-BN nanosheets based on the obtained potential parameters, the MD simulation is performed according to the following steps. Firstly, the conjugate gradient method is used to minimize the energy of the initially constructed bilayer h-BN nanosheet model. Then, the obtained energy-minimized bilayer h-BN nanosheet is relaxed at a constant temperature for 20 ps to reach an equilibrium state. In order to bring the entire system to an equilibrium and stable state as much as possible, the NVT ensemble and Nosé-Hoover thermostat are specially utilized to ensure that the number of atoms, volume, and temperature of the system remain constant. During the simulation, the Verlet algorithm is adopted to solve the motion equations, which has second-order accuracy in time and is widely used in MD simulations to improve computational efficiency and accuracy. Finally, depending on the specific simulation content, there may be differences in the subsequent steps, which will be further introduced in the respective sections for discussing different properties of the simulation.

## Results and discussion

In this section, we present the results of the simulations described earlier, using the chosen potential. The study extensively investigates the relationship between the tensile properties of bilayer h-BN nanosheets with the applied strain rate under five stacking modes, the differences in mechanical performance of h-BN when it is pulled along different crystal directions, and the effect of temperature on the tensile properties. Additionally, the vibration characteristics of the bilayer h-BN nanosheets in different stacking modes is also analyzed by the MD simulation and the theoretical method based on the nonlocal elasticity in detail.

### Strain rate effect

In this section, we mainly investigate the effect of different strain rates on the tensile properties of bilayer h-BN nanosheets in five different stacking modes along the armchair direction. It is well known that applying different strain rates during tensile testing may significantly affect the measured tensile properties of the material. Therefore, before studying the effects of various factors on tensile performance, we first explore whether different tensile strain rates will have an impact on the tensile properties. To examine this issue, we compare the stress–strain curves of the bilayer h-BN nanosheets under different strain rates ranging from 0.01 to 0.0005/ps, while being stretched along the armchair direction at a constant temperature of 300K.

**Figure 3:**
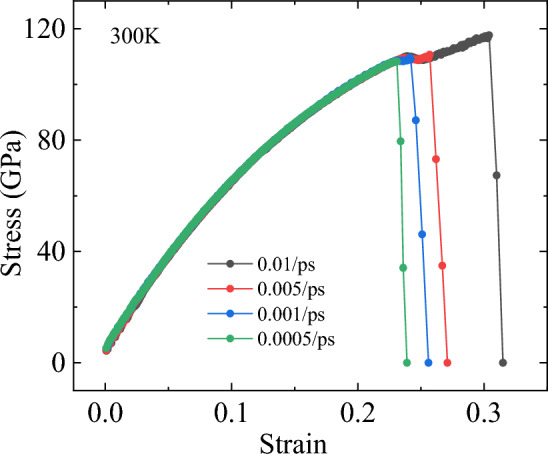
Stress–strain curve of h-BN-AA′ under different strain rates at 300 K along the armchair direction.

Figure 3 shows the stress–strain curves of the bilayer h-BN nanosheets of AA′ stacking mode under four different strain rates (0.01/ps, 0.005/ps, 0.001/ps, 0.0005/ps) when stretched along the armchair direction at an environmental temperature of 300K. It can be seen that the stress–strain curves of the AA′ stacking mode overlap significantly in elasticity stage, indicating that applying different strain rates does not affect the elastic modulus of the bilayer h-BN during stretching. Moreover, we can observe from Fig. 3 that the stress–strain curves only differ at the ultimate strain and ultimate stress, which refer to the maximum stress and strain that the bilayer h-BN nanosheet can reach when stretched, usually taken from the stress–strain curve just before fracture. This phenomenon is similar to the behavior of single-layer diamond under different strain rates during tensile deformation^38^. Because the results of the other four stacking modes are highly similar to that of the AA′ stacking mode, in order to make the article more concise, this section will not be further elaborated. The simulation results indicate that the elasticity properties of the bilayer h-BN are not significantly affected by the strain rate of 0.01/ps and there are only some differences in the ultimate stress and the ultimate strain. The effect on Young’s modulus is ignorable, which is suitable for the purpose of this research. We should note that the strain rate applied in the simulation is very large compared to the strain rate used in the laboratory. In this regard, recently developed meta-dynamics^39^ may be able to provide some additional insights into strain rate effects that warrant further investigation.

### Influence of crystal orientation

**Figure 4:**
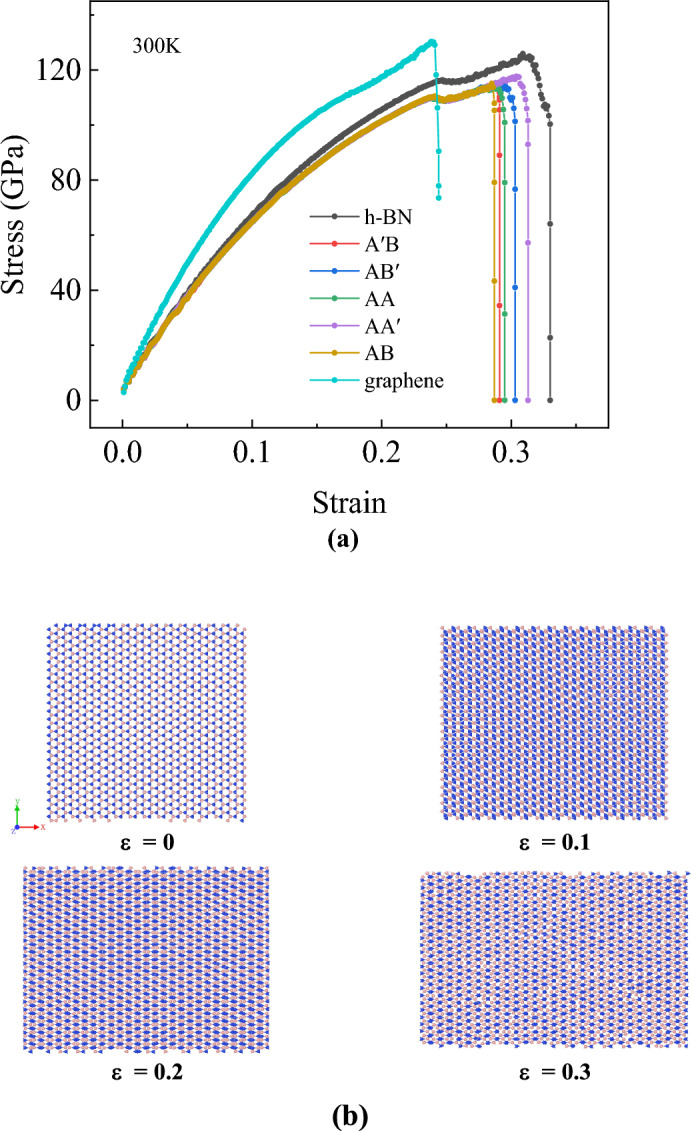
(**a**) Stress–strain curves of bilayer h-BN under five different stacking modes and monolayer h-BN and graphene along armchair direction at 300 K. (**b**) Atomic configurations for the tensile along armchair direction at different strain under AA′ stacking mode.

**Figure 5:**
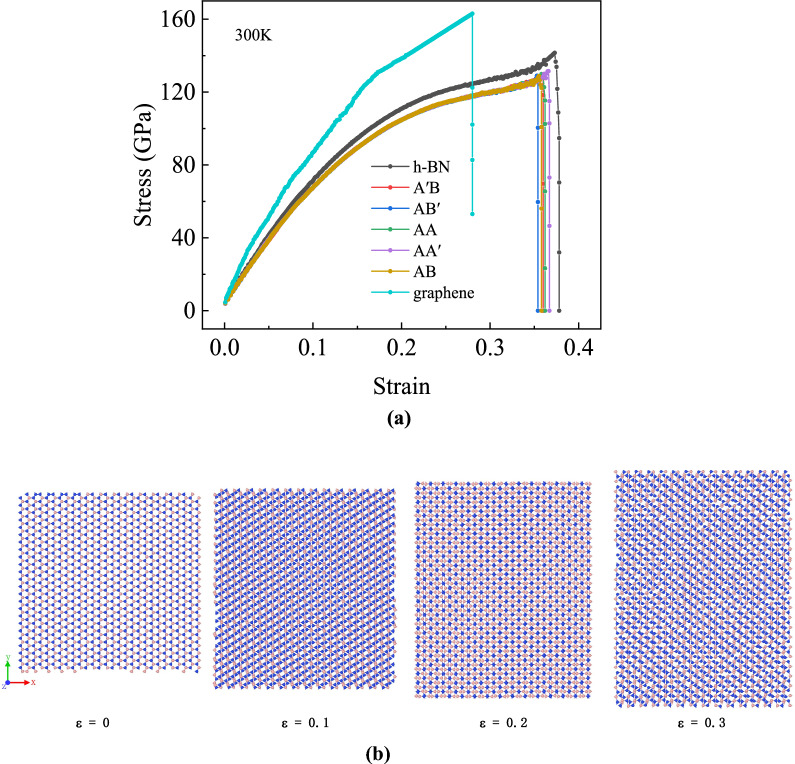
(**a**) Stress–strain curves of bilayer h-BN under five different stacking modes and monolayer h-BN along zigzag direction at 300 K. (**b**) Atomic configurations for the tensile along zigzag direction at different strain under AA′ stacking mode.

As the strain rate does not affect the elasticity properties of bilayer h-BN nanosheets showed in “Strain rate effect” Section, in this section, we study the performance of bilayer h-BN nanosheets stretched in different directions under five stacking modes at a temperature of 300 K and a strain rate of 0.01/ps. Moreover, we compare the Young’s modulus of bilayer h-BN in the five stacking modes along the armchair and zigzag directions with its parent material, monolayer h-BN^7^, and graphene^2^ as shown in Figs. 4a and 5a, respectively. The tensile data for single-layer h-BN and graphene in the simulation are consistent with existing studies^2,7^, indicating the reliability of the tensile simulation.

Figures 4 and 5 show the stress–strain curves of bilayer h-BN nanosheets in five stacking modes under uniaxial tension along the armchair and zigzag directions, respectively, and the corresponding structural evolution of AA′ stacking mode. In Fig. 4a when the bilayer h-BN nanosheets are stretched along the armchair direction, an interesting phenomenon can be observed. Firstly, the stress increases at a relatively low rate with the increase of strain at first. However, when the stress reaches a critical value of 110 GPa, it remains constant for a certain period of time while the strain continues to increase. As the nanosheet is stretched further along the armchair direction, the rate of stress increase gradually slows down, eventually reaching the ultimate stress and strain, which are approximately 116 GPa and 0.28, respectively. This indicates that the bilayer h-BN will experience ductile fracture when stretched along the armchair direction. Figure 4a also demonstrates that the ultimate stress of graphene is greater than h-BN, while the ultimate strain of graphene is lower than h-BN along armchair direction. Figure 4b presents morphology of tensile process, it can be seen that as the strain increases partial fracture occurs when the strain reaches nearly the ultimate strain 0.3.

In zigzag direction, the ultimate stress of graphene is larger than h-BN, whereas the ultimate strain of graphene is also greater than h-BN as shown in Fig. 5a. From Fig. 5a when the bilayer h-BN nanosheets are stretched along the zigzag direction, a completely different phenomenon is observed. The stress and strain both increase continuously, and when the stretching stress and strain reach a certain threshold, which are approximately 128 GPa and 0.36, respectively, both the stress and strain rapidly decrease, indicating brittle fracture of the bilayer h-BN nanosheets when stretched along the zigzag direction. Different from Fig. 4b that no fracture is observed in Fig. 5b when the strain is 0.3.

It can be observed that the Young’s modulus of monolayer h-BN is greater than that of bilayer h-BN, which is about 5%, regardless of the stretching in the direction of armchair or zigzag. This is because there is an atomic-level interlayer sliding process between the layers in multilayer materials^40–42^, and the interlayer deformation between the layers leads to differences in the Young's modulus between monolayer and multilayer structures. However, similarly, when the number of layers increases from monolayer to 8 layers, h-BN exhibits strong interlayer coupling, resulting in little change in the modulus^43^. This is because, when the thickness increases to 8 layers, a distinct multilayer structure is formed, enhancing the interactions between layers. This interlayer bonding hinders interlayer sliding and relative displacement, making multilayer hexagonal boron nitride more rigid under stress. Therefore, with an increase in the number of layers, the overall elastic properties are dominated by interlayer bonding. Additionally, monolayer h-BN and bilayer h-BN have different lattice structures and atomic arrangements, which influence the material’s stiffness and elastic properties. Monolayer h-BN has a hexagonal lattice structure, where each nitrogen and boron atom is connected to three neighboring atoms, forming a relatively stable structure. This structure typically exhibits a higher Young’s modulus, indicating that monolayer h-BN has higher stiffness. While the bilayer h-BN is formed by stacking two monolayer h-BN sheets in the vertical direction, resulting in a different lattice structure and a lower Young’s modulus. Additionally, intralayer van der Waals forces and Coulombic forces also contribute to a slightly lower Young’s modulus. Moreover, in the armchair tension case, only two out of every six B–N bonds in a unit cell are significantly loaded and stretched, while the other four bonds along the perpendicular direction of tension experience only fractional changes due to the conventional Poisson effect^38,44^. This results in different Poisson's ratios between single-layer and multi-layer configurations, contributing to the performance disparities between the two.

H-BN exhibits different fracture behaviors when stretched along different crystallographic directions, namely ductile fracture occurs along the armchair direction, while brittle fracture occurs along the zigzag direction. This is due to the differences in the crystal structure and bonding properties of h-BN crystals along different directions^45^. The reason for the occurrence of ductile fracture during stretching along the armchair direction is due to strong bonding and a significant presence of slip planes. Strong bonding imparts the material with high strength and ductility, while the presence of slip planes allows atoms to slide relatively easily along these planes, facilitating plastic deformation. The occurrence of brittle fracture in the zigzag direction is due to weak bonding and fewer slip planes, resulting in lower strength and ductility. The ductile fracture behavior of bilayer h-BN nanosheets along the armchair direction provides some insights for its potential applications in NEMS^45^. Ductile fracture allows bilayer h-BN nanosheets to withstand continuously increasing strains and deformations without sudden failure, which is beneficial for scenarios involving mechanical stresses or deformations that may occur during device operation. However, ductile fracture typically involves plastic deformation, which may result in a reduction of the stiffness and the ultimate strength of bilayer h-BN nanosheets.

It can be observed that the stress–strain curves for the same stretching direction almost overlap for different stacking patterns from Figs. 4a and 5a, with only slight differences in ultimate stress and strain. However, for the same stacking mode, the stress–strain curves for stretching along the armchair and zigzag directions are not the same. This result indicates that different crystal directions have a significant effect on the tensile properties of bilayer h-BN nanosheets, while the stacking mode has a relatively small effect on their tensile properties.

**Figure 6:**
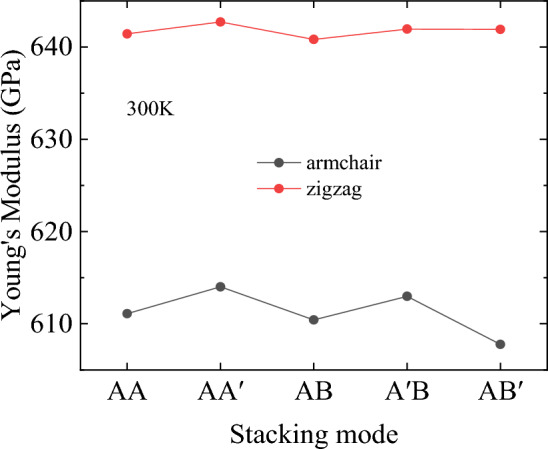
Young’s modulus of bilayer h-BN nanosheets under different stacking modes at 300 K.

**Figure 7:**
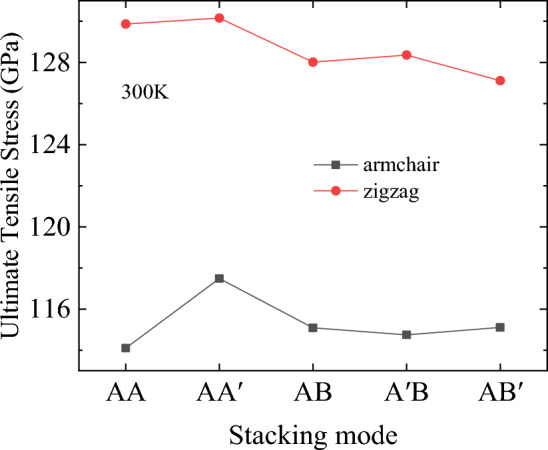
Ultimate tensile stress of bilayer h-BN nanosheets under different stacking modes at 300 K.

**Figure 8:**
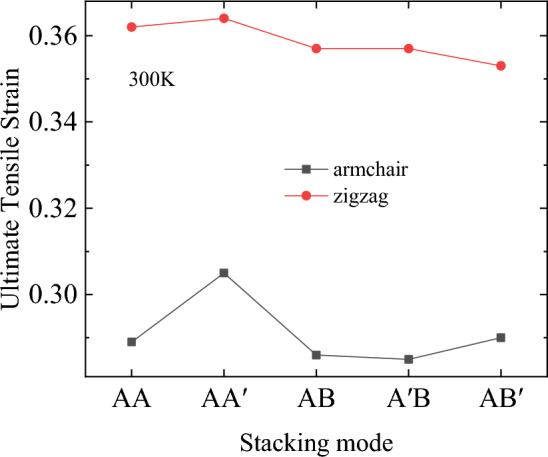
Ultimate tensile strain of bilayer h-BN nanosheets under different stacking modes at 300 K.

Based on the stress–strain curves obtained from the above tensile simulations, the Young’s modulus of bilayer h-BN nanosheets along different crystallographic directions in different stacking modes are calculated. When calculating Young’s modulus, the strain ranges from 0.01 to 0.11 on the stress–strain curve is considered as the linear elastic region, and the slope of the linear portion is used to characterize the Young's modulus. The relationships between the ultimate stress, ultimate strain, stacking modes, and crystallographic direction are presented by line charts as shown in Figs. 6, 7 and 8.

The Fig. 6 demonstrates that stretching along different crystal directions can affect the Young’s modulus of bilayer h-BN. The Young’s modulus is about 640 GPa when the h-BN nanosheet is stretched along the zigzag direction, whereas the Young’s modulus is only about 610 GPa when it is stretched along the armchair direction. The difference of Young's modulus in the armchair and zigzag directions is about 30 GPa, indicating that bilayer h-BN is highly anisotropic. This is consistent with the characteristics of the crystal structure, because the hexagonal structure is usually responsible for the anisotropy of the material, where the difference is greatest along the armchair and zigzag directions. In graphene, this anisotropy is usually ignored because the material is all carbon atoms. However, in the case of bilayer h-BN materials, the structure contains two different atoms (B and N), so anisotropy must be taken into consideration when it comes to theoretical analysis. The above results are consistent with the conclusions on the study of diamond and graphene^45^, indicating that the crystal orientation has a significant impact on the tensile properties of 2D hexagonal structures^38^.

It is further proved that the stacking modes have little effect on the tensile properties by Figs. 7 and 8. For example, under all the five stacking modes, the ultimate stress of stretching along the zigzag direction is about 128 GPa, which is 10% higher than the 116 GPa of stretching along the armchair direction. Moreover, the ultimate strain of stretching along the zigzag direction is about 0.36, which is 20% higher than that of stretching along the armchair direction, which is approximately 0.29.

Furthermore, Poisson’s ratios along armchair and zigzag directions denoted respectively by *v*_*xy*_ and *v*_*yx*_ at 300 K are calculated based on the above tensile simulations. Stretching along the zigzag direction has a Poisson’s ratio of about 0.32 for the five different stacking modes, which is 31% larger than stretching along the armchair direction with a Poisson's ratio of 0.22. As expected, the crystal orientation also has a significant effect on Poisson's ratio, while the different stacking patterns have little effect on Poisson’s ratio.

All Figs. 6, 7 and 8 and Table 2 show that the strong anisotropy of bilayer h-BN and the stacking modes have little influence on the tensile properties. However, by carefully observing the comparison of five stacking modes, we can find that Young’s modulus, ultimate stress, ultimate strain and Poisson’s ratio are higher in AA′ stacking mode than in the other four stacking modes.

**Table 2:** Poisson's ratio in different stacking modes at 300 K.

	AA	AA′	AB	A′B	AB′
*v*_*xy*_ (armchair)	0.3328	0.3912	0.3637	0.3220	0.3272
*v*_*yx*_ (zigzag)	0.1839	0.2529	0.2273	0.2295	0.2217

Although stacking mode has little effect on tensile properties, AA′ stacking mode still has the best tensile properties among the five different stacking modes. The reason for this result can be attributed to its the least ground state energy^46^ and can be separated directly from the naturally occurring bulk h-BN crystals^11^. The bilayer h-BN in the AA′ stacking mode exhibits the lowest ground state energy, indicating that the system is in its most stable state, corresponding to the most stable configuration of the boron nitride crystal. In this scenario, the crystal structure is more robust and ordered, contributing to the overall stability of the material. The least ground state corresponds to the minimum potential energy and minimum internal energy state of the system, indicating relatively weak interaction forces between atoms. Molecules and atoms in the system tend to be in a more stationary state, making the material more resistant to external deformations and reducing the likelihood of deformation occurring. Additionally, the least ground state corresponds to the state of the system with the lowest vibrational energy, implying that atomic oscillations near their equilibrium positions are minimal, further diminishing the tendency for deformation under external forces. Therefore, in tension, the bilayer h-BN in the AA′ stacking mode exhibits the highest values of elastic modulus along with superior resistance to deformation.

### Shear behavior

**Figure 9:**
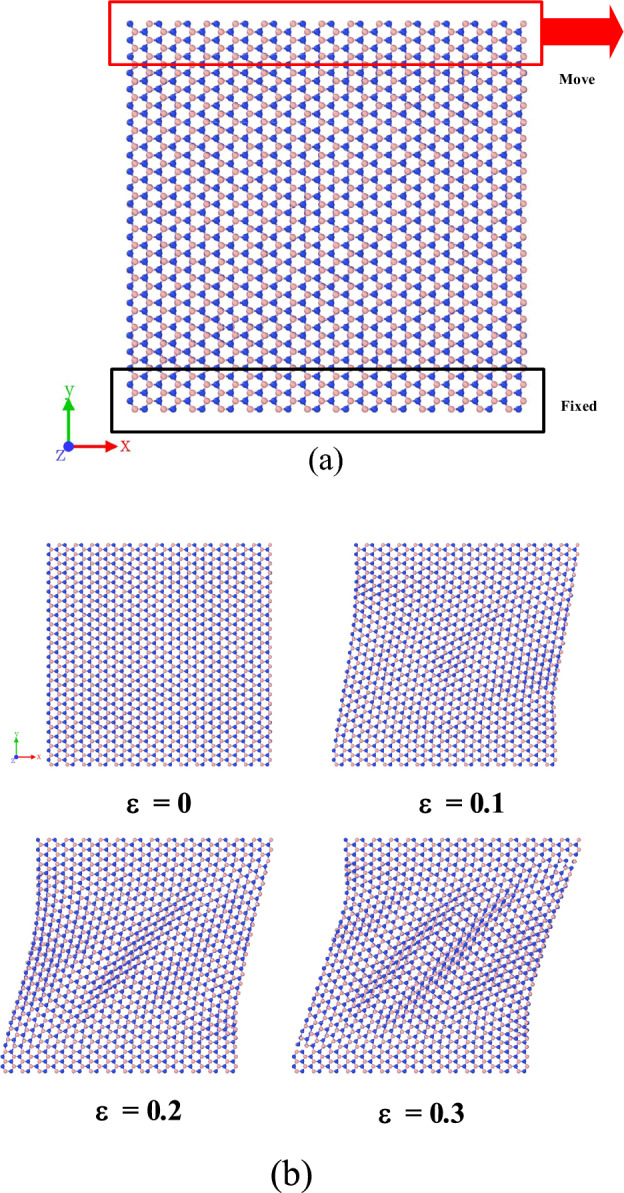
(**a**) Atoms in fixed group are fixed while a uniaxial tension is applied to stretch the atoms in move group. (**b**) Atomic configurations for the shear of bilayer h-BN-AA′ under 300 K.

This section mainly focuses on the shear modulus under five stacking modes. Although the five stacking modes previously obtained have little influence on the tensile properties, and AA′ shows the best tensile properties under the five stacking modes. Therefore, before the shear simulation, such similar pattern may occur in shear properties. During the shearing process, the bilayer h-BN nanosheet is divided into three groups, namely top, middle and bottom, as shown in the Fig. 9a. The uniaxial stretching is applied to the atoms in the top group while the atoms in the bottom group are fixed and the atoms in the middle group are then held in place. Through this simulation, the shear modulus of the bilayer h-BN nanosheets under five stacking modes can be calculated at the temperature of 300 K. The shear process of double-layer h-BN nanosheet is shown in Fig. 9b.

Similarly, the stage of strain between 0.01 and 0.11 in the stress–strain curve is regarded as the elasticity stage, and the shear modulus is calculated at this stage. Through MD simulation, the shear modulus is obtained as shown in Table 3. Just as we assumed, the stacking mode has little effect on the shear performance, and the difference between the maximum and minimum shear modulus is 8.14%. However, under the five stacking modes, the shear modulus of AA′ stacking mode is the largest, which is 117 GPa. All the above results show that AA′ stacking mode still shows the best tensile and shear properties in all five stacking modes.

**Table 3:** Shear modulus in different stacking modes at 300 K.

Stacking Mode	Shear modulus *G*_*xy*_ (GPa)
AA	112.5259
AA′	116.9785
AB	107.6783
A′B	111.1933
AB′	107.4555

### Influence of temperature

In fact, it is well known that temperature is one of the key factors affecting the mechanical properties of nanomaterials. H-BN, commonly known as “white graphite”, with excellent heat dissipation and insulation performance, has good thermal conductivity and is expected to be a new generation of head dissipation materials for electronic chips. Therefore, in this section, the influence of temperature on the tensile properties of bilayer h-BN is studied. According to previous research results, AA′ exhibits the best tensile and shear properties under five stacking modes. Therefore, in this section, we carry out MD simulation only for AA′ stacking mode, and study the influence of temperature on anisotropic materials once again. The main parameters of the simulation are as follows: the strain rate of tensile deformation is kept at 0.01/ps, and the bilayer h-BN nanosheets are set at different temperatures to be stretched along the armchair and zigzag directions, respectively, to obtain the stress–strain curves. The simulated temperature starts from 100 K and increases to 2100 K with 200 K as a step.

**Figure 10:**
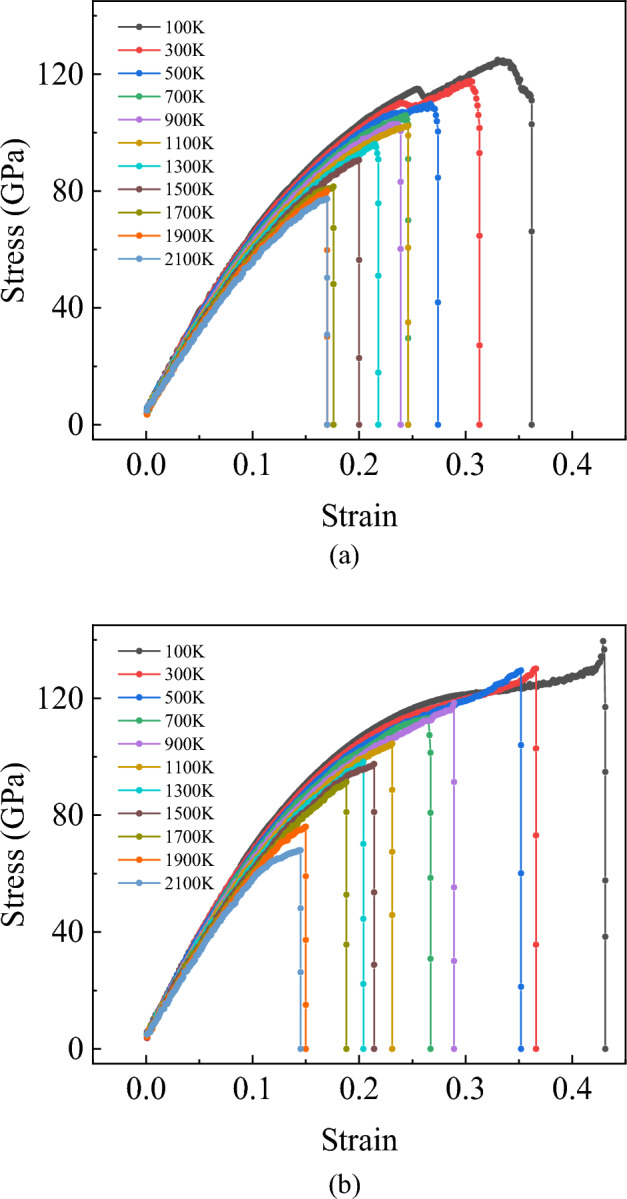
Stress–strain curves of bilayer h-BN-AA′ nanosheet along the armchair (**a**) and zigzag (**b**) direction under the temperature ranging from 100 to 2100 K.

Figure 10 records the relationship between the temperature and the stress–strain curve of the bilayer h-BN nanosheets in AA′ stacking mode along the armchair and zigzag directions. The ultimate stress and strain are all decrease with the increase of the temperature for the both directions. An interesting observation is that, the yield stage is observed shortly only when the temperature is less than or equal to 500 K along armchair direction.

**Figure 11:**
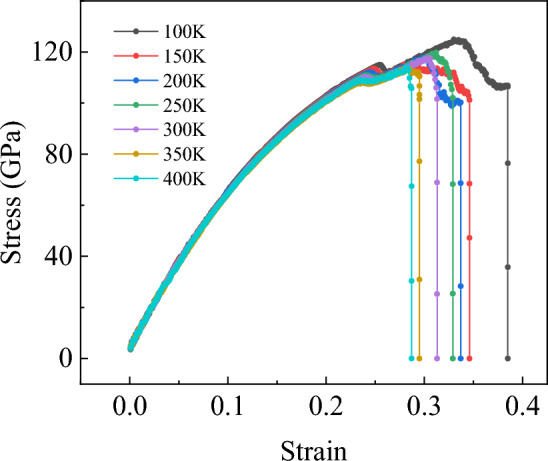
Stress–strain curves of bilayer h-BN-AA′ nanosheet along the armchair direction under the temperature ranging from 100 to 400 K.

Since the previous temperature gradient is 200 K, it is not easy to determine the specific temperature range of the yield phenomenon. Therefore, the temperature simulation of the yield phenomenon when stretching along the armchair direction is further developed. This time, the temperature gradient is set to 50 K, and the temperature from 100 to 400 K is chosen, totaling 7 sets of data. In Fig. 11, it can be clearly observed that when the temperature rises to about 400 K, there is almost no yield phenomenon. The results show that the yield of the bilayer h-BN nanosheets can be observed only when the temperature is below 400 K and the tension is stretched along the armchair direction. The reason for the yield phenomenon is the slip of the inner crystals during the tensile deformation of the bilayer h-BN nanosheets. When sliding to a certain extent, dislocation density between crystals will gradually increase until plastic deformation occurs. At this time, the strength of the bilayer h-BN nanosheet will gradually decrease, forming a stress drop point, namely the yield point.

**Figure 12:**
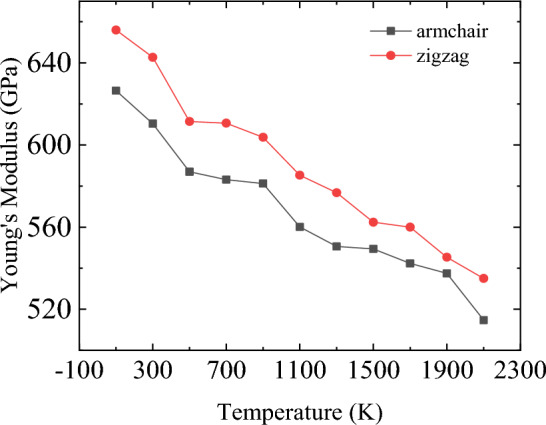
Temperature dependence of Young’s modulus obtained for bilayer h-BN-AA′ nanosheets.

**Figure 13:**
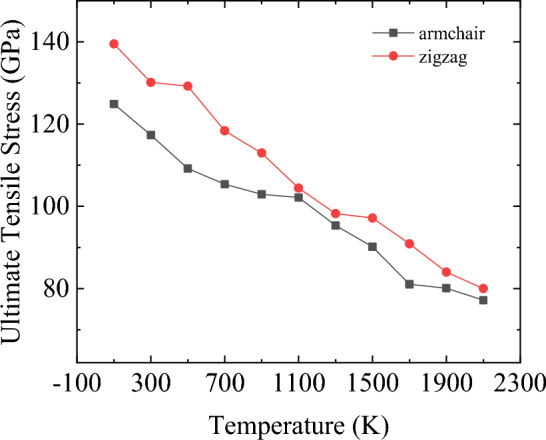
Temperature dependence of the ultimate tensile stress obtained for bilayer h-BN AA′ nanosheets.

**Figure 14:**
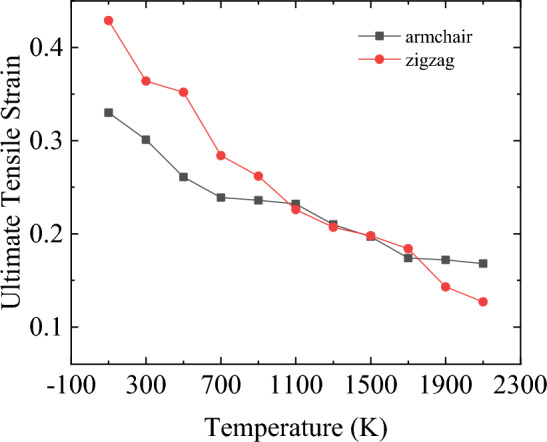
Temperature dependence of the ultimate tensile strain obtained for bilayer h-BN AA′ nanosheets. The strain rate is kept constant at 0.01/ps.

Furthermore, according to the stress–strain curve recorded in the Fig. 10, Young’s modulus, the ultimate stress and ultimate strain of bilayer h-BN nanosheets in AA′ stacking mode are also calculated along the armchair and zigzag directions when the temperature rises from 100 to 2100 K, as shown in the following Figs. 12, 13 and 14.

It can be seen from Fig. 12 that Young’s modulus decreases with the increase of temperature when stretching in both the armchair and the zigzag directions, and the average rate of change is almost 60 MPa/K. Moreover, when the temperature is the same, the Young’s modulus of stretching along the zigzag direction is larger than that along the armchair direction, with a gap of about 30 GPa. This shows that the tensile properties of the bilayer h-BN nanosheets are changed by temperature with the chirality, which is very similar to the results observed in the single-layer h-BN nanosheets^9^.

With the increase of temperature, the ultimate stress in both directions also shows a decreasing trend in Fig. 13. As the temperature increases from 100 to 2100 K, the ultimate stress in stretching along the armchair direction decreases by about 50 GPa and in the zigzag direction by 70 GPa. However, as the temperature is less than 1100 K, the effect of chirality on the ultimate stress is obvious, and the difference of the ultimate stress in the armchair direction and the zigzag direction can reach 20 GPa at most. When the temperature is higher than 1100 K, the effect of chirality on the ultimate stress is little, and there is no significant difference in the ultimate stress when stretching along the two directions.

Different from the law presented by Young’s modulus and ultimate stress, the variation trend of ultimate strain in Fig. 14 is more complex. When the temperature is less than 1100K, the ultimate strain along the zigzag direction is greater than that along the armchair direction. When the temperature reaches between 1100 and 1700 K, the ultimate strain along both the two directions are nearly same. However, when the temperature is greater than 1700 K, this trend is reversed, and then the ultimate strain along the armchair direction is greater than that along the zigzag direction. However, by observing the curve of ultimate strain variation along the two directions of stretching in Fig. 14, it can be clearly seen that the reduction rate of ultimate strain along the zigzag direction with the change of temperature is much greater than that in the armchair direction. This can explain why the ultimate strain in the armchair direction is greater than that in the zigzag direction at high temperature, indicating that the effect of temperature on the ultimate strain in the armchair direction is less than that in the zigzag direction.

From Figs. 12, 13, and 14, it can be observed that as the temperature increases, the Young's modulus and strength of bilayer h-BN show a clear decreasing trend. This phenomenon is primarily attributed to four main reasons. Firstly, with the increase in temperature, the initial ground state energy of bilayer h-BN rises. The higher ground state energy makes the system more unstable, reducing its ability to resist deformation. Secondly, elevated temperatures result in larger thermal vibrations of atoms within the system, leading to increased oscillation amplitudes near their equilibrium positions. This, in turn, increases the average atomic spacing within the system. Since Young’s modulus is related to the atomic spacing, this leads to a decrease in Young’s modulus. Additionally, high temperatures induce thermal expansion in the system, making deformation more likely under tensile loading, consequently reducing the material's strength. Lastly, at high temperatures, the activity of thermal excitations and dislocation motion increases, causing changes in the local structure within the crystal and subsequently affecting the overall Young’s modulus and strength.

### KC potential

**Figure 15:**
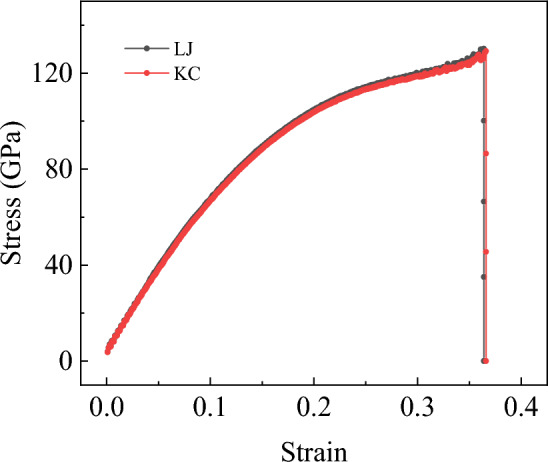
The tensile stress–strain curves for bilayer h-BN in the AA′ stacking mode under two different potentials.

The previous sections extensively investigated the tensile and shear properties of bilayer h-BN in five different stacking modes, revealing that the bilayer h-BN in the AA′ stacking mode exhibits the most superior tensile and shear properties. This section focuses on the bilayer AA′ h-BN and explores the differences in its tensile and shear properties when using the KC potential compared to previous results. Along the zigzag direction, we compare the tensile results under two different potentials, as shown in Fig. 15.

Similarly, we calculate the Young’s modulus, the ultimate stress and the ultimate strain, and compare the results with those obtained under the LJ potential, as summarized in Table 4. We found that the difference between the two potentials was only 1.3%, indicating that using the LJ potential to characterize the interlayer van der Waals forces in bilayer h-BN is appropriate for tensile simulation.

**Table 4:** The performance parameters during the tensile process of bilayer h-BN under the LJ and KC potentials.

Potential	Young’s modulus (GPa)	Ultimate stress (GPa)	Ultimate strain
LJ	642.7115	130.16005	0.364
KC	634.3768	129.16881	0.366

**Figure 16:**
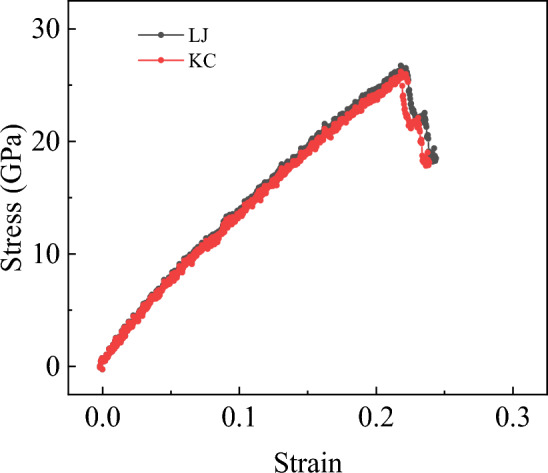
The shear stress–strain curves for bilayer h-BN in the AA′ stacking mode under two different potentials.

To reduce the randomness of the simulation results, we further utilized the KC potential for shear simulation, and obtained the shear stress–strain curves under both potentials. As shown in Fig. 16, the results revealed that the difference between the two potentials was also only 2% during the shear process. In summary, when the allowed difference is within 3%, we can conclude that the LJ and KC potentials yield almost identical results for characterizing the interlayer van der Waals forces in bilayer h-BN during tensile and shear progress.

### Natural frequency

**Figure 17:**
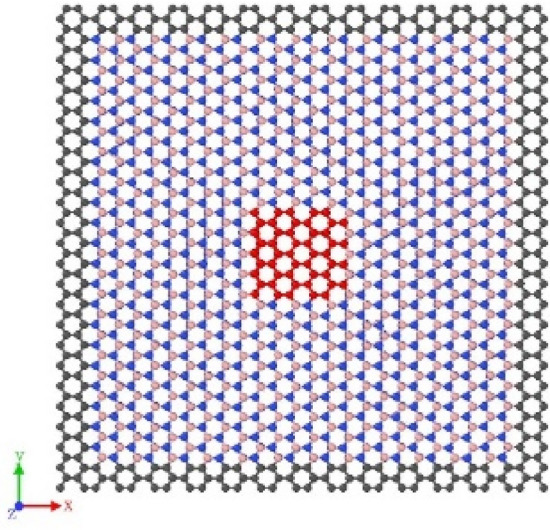
The boundary conditions initially set in the model in MD simulations. Atoms in gray are simply supported all the time and an initial displacement is applied in the group of red atoms.

In the previous sections, the LJ potential was used to describe the interlayer van der Waals forces in bilayer h-BN to study its tensile and shear properties. In this section, the vibration behavior of bilayer h-BN nanosheets under different stacking modes is investigated in order to further understand the potential application of bilayer h-BN serve in resonators. Since the research by V. Lebedeva et al. has shown that the KC potential function can account for relative translations between layers^35^, this section employs the KC potential for the free vibration simulation of bilayer h-BN. The MD simulations are performed to simulate the vibration behavior of bilayer h-BN nanosheet along the *z* direction which is vertical to the nanosheet plane, as shown in Fig. 17. The atoms of the bilayer h-BN nanosheet are grouped, the gray atoms group in the outermost portion of the nanosheet is simply supported, and an initial displacement is applied to the red atoms group in the middle of the nanosheet.

**Figure 18:**
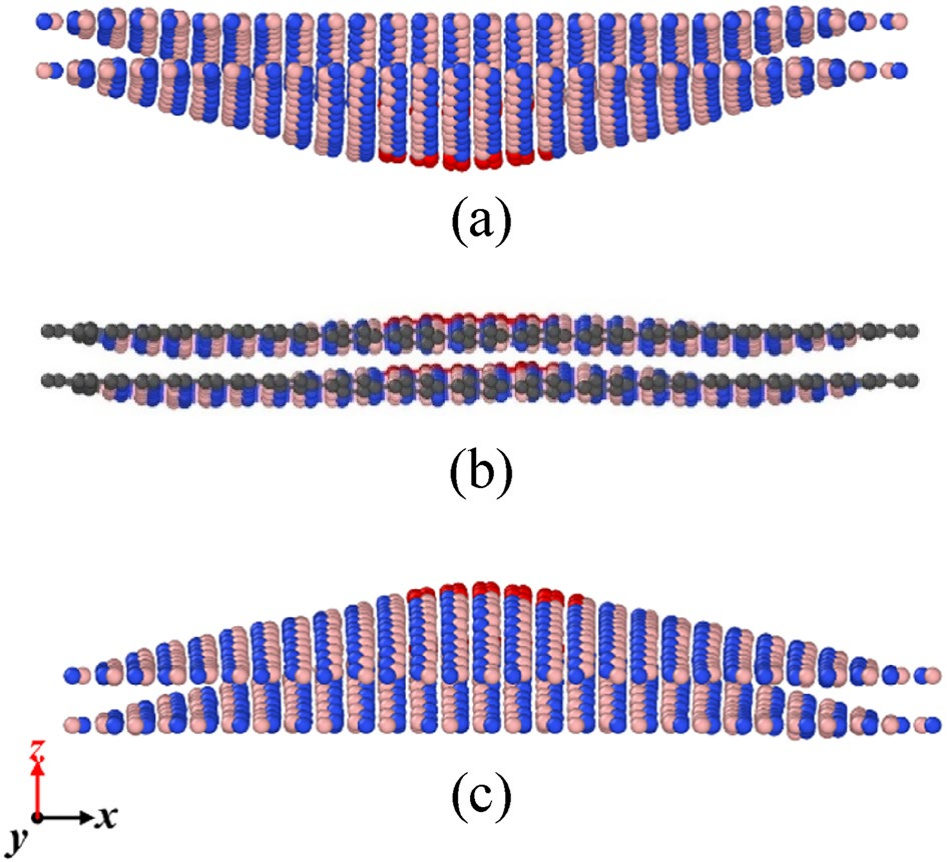
A period of free vibration diagrams of bilayer h-BN nanosheets.

**Figure 19:**
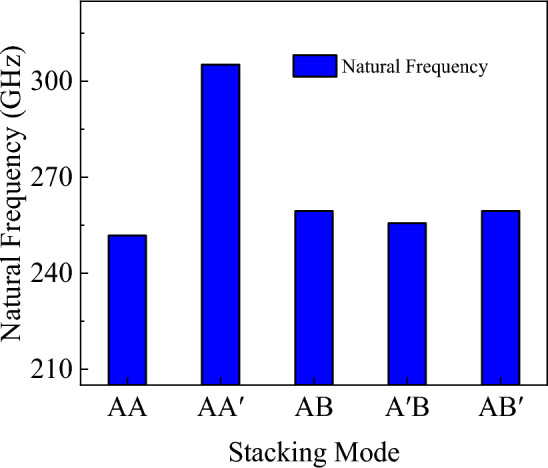
The natural frequency of bilayer h-BN nanosheets under five different stacking modes.

**Figure 20:**
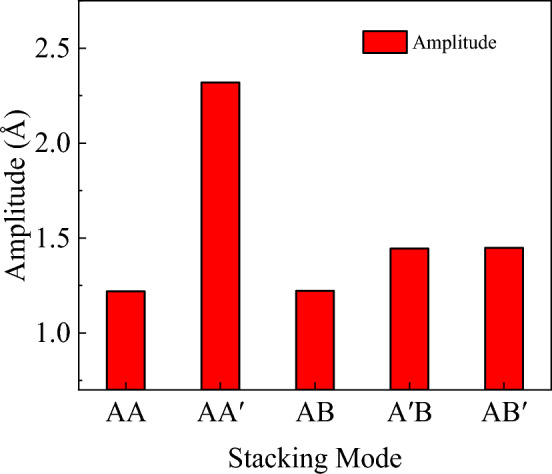
The amplitude of bilayer h-BN nanosheets under five different stacking modes.

The nanosheet can then vibrate freely as shown in Fig. 18, recording their displacement of red atoms group in the *z* direction during the free vibration. According to the obtained time and displacement, code is written in MATLAB to obtain the natural frequencies of bilayer h-BN under five stacking modes using fast Fourier transform (FFT) method, as shown in Figs. 19 and 20.

**Figure 21:**
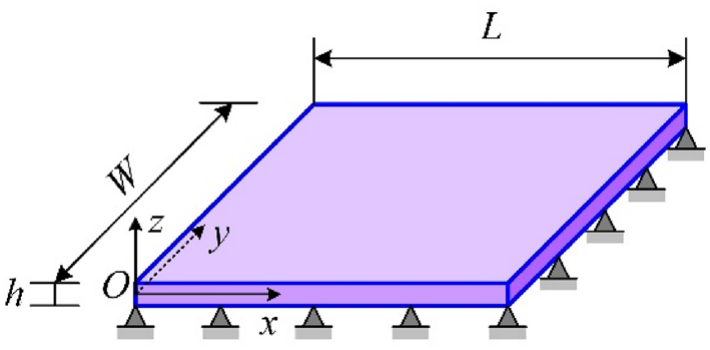
Diagrammatic sketch of single-orthotropic h-BN nanoplate.

The above data are obtained through MD simulations, and the analytical theory is utilized to verify the accuracy of simulation results. We regard the bilayer h-BN nanosheets as single layer orthogonal anisotropic plates, as shown in Fig. 21. The length *L* of the nanoplate in the *x* direction is 6 nm, the width *W* in the *y* direction is 6 nm, and the thickness *h* in the *z* direction is 0.333 nm.

At the nanoscale, the interactions between atoms and grain boundaries in the crystal and the in-plane slip of bilayer h-BN nanosheets can also significantly influence on the vibration properties of nanomaterials. Therefore, in order to describe the vibrational behaviors of bilayer h-BN nanosheet more accurately, the nonlocal elastic theory is applied and in the nonlocal elasticity theory, the elastic parameters of materials are adopted from the previous MD simulation. Referring to the vibration analysis of double-layer orthogonal anisotropic nanoplates based on nonlocal theory presented by Pouresmaeeli^47^, the motion differential equation of single-layer biorthogonal anisotropic h-BN nanoplate can be obtained as follows:

5 $$ \begin{gathered} D_{11} \frac{{\partial^{4} w}}{{\partial x^{4} }} + 2\left( {D_{12} + 2D_{66} } \right)\frac{{\partial^{4} w}}{{\partial x^{2} \partial y^{2} }} + D_{22} \frac{{\partial^{4} w}}{{\partial y^{4} }} + I_{0} \frac{{\partial^{2} w}}{{\partial t^{2} }} - I_{2} \left( {\frac{{\partial^{4} w}}{{\partial x^{2} \partial t^{2} }} + \frac{{\partial^{4} w}}{{\partial y^{2} \partial t^{2} }}} \right) \\ + \mu^{2} \nabla^{2} \left[ {I_{2} \left( {\frac{{\partial^{4} w}}{{\partial x^{2} \partial t^{2} }} + \frac{{\partial^{4} w}}{{\partial y^{2} \partial t^{2} }}} \right) - I_{0} \frac{{\partial^{2} w}}{{\partial t^{2} }}} \right] = 0, \\ \end{gathered} $$

where *w* denote displacement of the point (*x,y,*0) along *z* directions, *D*_11_, *D*_12_, *D*_22_ and *D*_66_ denote the bending rigidity of the orthotropic nanoplate and they are defined as

6 $$ \begin{gathered} D_{11} = \frac{{Y_{x} h^{3} }}{{12\left( {1 - v_{xy} v_{yx} } \right)}},D_{12} = \frac{{v_{xy} Y_{y} h^{3} }}{{12\left( {1 - v_{xy} v_{yx} } \right)}} \hfill \\ D_{22} = \frac{{Y_{y} h^{3} }}{{12\left( {1 - v_{xy} v_{yx} } \right)}},D_{66} = \frac{{G_{xy} h^{3} }}{12}. \hfill \\ \hfill \\ \end{gathered} $$

here* Y*_*x*_ and *Y*_*y*_ denote the Young’s moduli in armchair and zigzag directions, respectively. *G*_*xy*_ is the shear modulus,* v*_*xy*_ and* v*_*yx*_ are Poisson’s ratios along armchair and zigzag directions, respectively. All these parameter values are all acquired from MD simulations before. The Laplace operator in the Cartesian coordinate system is given by $\nabla^{2} = \frac{{\partial^{2} }}{{\partial x^{2} }} + \frac{{\partial^{2} }}{{\partial y^{2} }}$, and the parameter *μ* ($\mu = e_{0} a$) is the scale coefficient revealing the small-scale effect on responses of structures in the nanosize. The scale coefficient *μ* is related to the size and length of the model, which can only be determined by experiment or calculation, in this paper $\eta = \frac{\mu }{L}$ and $\eta$ is two time the thickness of bilayer h-BN.

But the above given equations are general without considering the boundary conditions. Combined with the conditions set in the simulation process, four sides of the nanoplate model are simply supported and the displacement of the four sides of the nanoplate are zero. Under such conditions, Navier method is adopted to solve the Eq. (5), and the displacement solution is defined as follows:

7 $$ w = \sum\limits_{m = 1}^{\infty } {\sum\limits_{n = 1}^{\infty } {W_{mn} \sin \left( {\frac{{m{\uppi }}}{L}x} \right)\sin \left( {\frac{{n{\uppi }}}{W}y} \right)e^{{i\omega_{mn} t}} } } , $$

By substituting Eq. (7) into the Eq. (5), the natural frequencies of single layer orthotropic nanoplates can be obtained as follows:

8 $$ \omega_{mn} = \frac{{D_{11} \left( {\frac{{m{\uppi }}}{L}} \right)^{4} + 2\left( {D_{12} + 2D_{66} } \right)\left( {\frac{{m{\uppi }}}{L}} \right)^{2} \left( {\frac{{n{\uppi }}}{W}} \right)^{2} + D_{22} \left( {\frac{{n{\uppi }}}{W}} \right)^{4} }}{{\rho h\left[ {1 + \left( {\frac{{h^{2} }}{12} + \mu^{2} } \right)\left( {\left( {\frac{{m{\uppi }}}{L}} \right)^{2} + \left( {\frac{{n{\uppi }}}{W}} \right)^{2} } \right) + \frac{{h^{2} }}{12}\mu^{2} \left( {\left( {\frac{{m{\uppi }}}{L}} \right)^{2} + \left( {\frac{{n{\uppi }}}{W}} \right)^{2} } \right)^{2} } \right]}}, $$

where *m* and *n* represent number of half wave and they are arbitrary positive integer. *W*_11_ and $\omega_{11}$ are the base exact amplitude and fundamental angular frequency solutions, respectively. We define the theoretical fundamental natural frequency as $f_{{{\text{Theory}}}} { = }{{\omega_{11} } \mathord{\left/ {\vphantom {{\omega_{11} } {2{\uppi }}}} \right. \kern-0pt} {2{\uppi }}}$ with unit GHz.

Up to now, the fundamental natural frequencies of bilayer h-BN nanosheets under different stacking modes are simulated by MD simulation and are analyzed based on nonlocal elastic theory. In order to facilitate the comparison between simulation results and theoretical analysis results, we summarized the results obtained by the two methods in the Table 5, and recorded the error between the two results. Moreover, the error results are given by $error{ = }\frac{{\left| {\left( {f_{{{\text{Theory}}}} - f_{{{\text{MD}}}} } \right)} \right|}}{{f_{{{\text{MD}}}} }}$.

**Table 5:** Comparisons of fundamental natural frequencies for bilayer h-BN nanosheets in different stacking modes.

	AA	AA′	AB	A′B	AB′
*f*_MD_ (GHz)	251.771	305.177	259.4	255.586	259.4
*f*_Theory_ (GHz)	245.892	289.359	248.667	245.254	254.358
*Error* (%)	1.589	5.183	4.138	4.042	1.944

As can be seen from Table 5, the fundamental natural frequencies of the five stacking modes obtained by MD are all in the range of 240 GHz and 305 GHz, and the corresponding frequencies predicted by theory are in the range of 245 GHz and 290 GHz. The maximum error of theoretical calculation and simulation results of the fundamental natural frequency of bilayer h-BN nanosheets in different stacking modes is only 5.183%. However, the largest error can be observed in the AA′ stacking pattern, which may be due to its reverse-stacked structure. The B atoms in one layer are directly above or below the N atoms in the adjacent layer, and vice versa. However, the results of this study are accurate enough, which means that the natural angular frequencies of the bilayer h-BN nanosheets in different stacking modes can be well predicted using the exact theoretical solution in Eq. (5) on the basis of the non-local theory.

It should be noted that the governing equation Eq. (5) along with Eq. (6) applies to the free vibration of bilayer h-BN nanosheets under any boundary condition. However, the natural frequency provided in Eq. (8) is specifically applicable to the case of free vibration with four sides simply supported. For other boundary conditions, the mode shape function given by Eq. (7) will vary. By selecting the appropriate mode shape function, similar procedures can also yield the corresponding natural frequencies. In summary, both our theoretical and MD methods can serve as references for bilayer h-BN nanosheets with general boundary conditions.

By selecting the appropriate mode shape function, similar procedures can also yield the corresponding natural frequencies. For MD simulations with other boundary conditions, similar approaches can be employed to obtain results. In summary, both our theoretical and MD methods can serve as references for bilayer h-BN nanosheets with various boundary conditions.

Since this theoretical model is applicable for solving problems with four-sided simply supported boundary conditions. Therefore, in order to provide a more detailed insight into the vibrational characteristics of bilayer h-BN under the five stacking modes and to reveal the complete physical phenomena, further investigations are carried out to examine the trends in the natural frequencies of bilayer h-BN under different sizes and with both ends simply supported boundary conditions.

**Figure 22:**
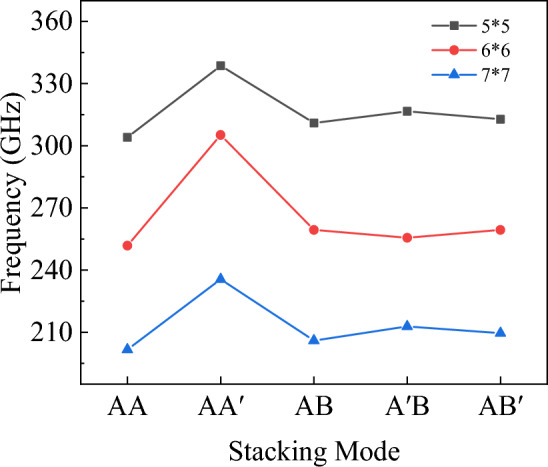
The natural frequencies of bilayer h-BN under five stacking modes with four sides simply supported conditions at different sizes (5 × 5, 6 × 6, 7 × 7).

Following the previous simulation procedures, the natural frequencies of bilayer h-BN with under five different stacking modes with three different sizes are initially investigated as shown in Fig. 22. The three sizes are 5 × 5, 6 × 6, and 7 × 7 nm, respectively and they are all under the boundary condition of four sides simply supported. In Fig. 22, the natural frequencies of bilayer h-BN decrease with increasing size for all stacking modes, which is primarily attributed to quantum size effects^16,29,48^. The quantum size effect can lead to changes in the energy levels of electrons and phonons. As the size of bilayer h-BN increases, the quantum size effect gradually diminishes, resulting in a decrease in natural frequencies.

**Figure 23:**
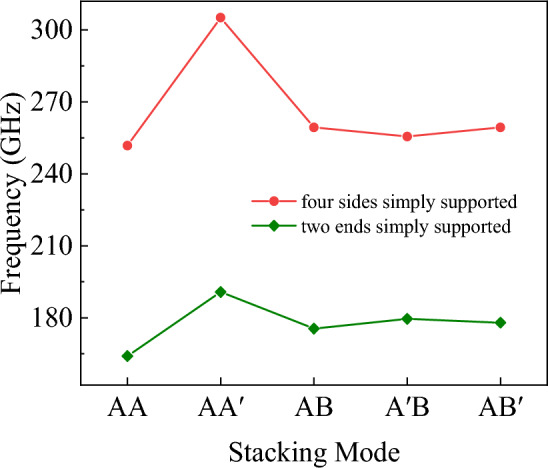
The natural frequencies of bilayer h-BN under five stacking modes with different boundary conditions but at the same size (6 × 6).

The natural frequencies of bilayer h-BN with the same sizes (6 × 6) but different boundary conditions are investigated in Fig. 23. For the same size and stacking mode, the natural frequencies are higher when under four sides simply supported boundary conditions compared to two ends simply supported conditions (7%). This difference results from the stronger constraints of four-side support, which lead to higher vibration frequencies. In structures with two ends simply supported boundaries, the constraint of the boundary with two sides free is weak, which reduces stiffness and natural frequencies. Additionally, the constraint strength significantly influences difference of vibrational frequencies among different stacking modes, for example, the maximum difference between the five stacking modes is 4% with four sides simply supported and 7% with two ends simply supported. In summary, the weaker is the constrain, the greater difference are the frequencies between AA′ and AA stacking modes.

However, from Fig. 23, we can also observe that for the BN with two ends, the differences of frequencies among the five stacking modes are approximately within 7%. Upon closer examination of the differences in natural frequencies among the five stacking modes, it can be observed that the AA′ stacking mode has the highest natural frequencies, the AA stacking mode exhibits the lowest frequencies, the AB, A′B, and AB′ stacking modes have similar natural frequencies. The reason for this phenomenon is due to the differences in symmetry and atomic charge characteristics among the different stacking modes. The AA stacking mode is a stacking arrangement characterized by high periodicity and symmetry, where each layer fully overlaps with its adjacent layer, and each nitrogen atom is directly above another nitrogen atom, as are the boron atoms. This arrangement results in atomic layers with similar charge distributions, leading to repulsive forces between adjacent layers during vibrations, which affects the natural frequencies. In the AA′ stacking mode, although it also exhibits high periodicity and symmetry, the atoms in adjacent layers are different, and there are attractive forces between these different atoms, resulting in the highest natural frequencies. As for the AB, A′B, and AB′ stacking modes, the presence of some atomic displacements between different types of atoms leads to lower symmetry. However, due to the relatively small structural differences among these stacking modes, their natural frequencies are relatively close to each other.

## Conclusions

In conclusion, the mechanical properties including tensile, shear and Poisson’s ratio of bilayer h-BN nanosheets for armchair and zigzag directions at different strain rates and different temperatures in five different stacking modes are studied based on MD simulations. For analysis of the free vibration of the bilayer h-BN nanosheets, the governing equation of single orthotropic nanoplate is formulated based on the nonlocal theory. The natural frequency has been obtained analytically and by MD simulations. The following results are obtained.

1. The results demonstrate that the Young's modulus, Poisson's ratios, and shear modulus of bilayer h-BN nanosheets at 300 K exhibit higher values along the zigzag direction compared to the armchair directions. This indicates that bilayer h-BN nanosheets in different stacking modes are essentially anisotropic materials, with significant variations in tensile strength and tensile strain depending on crystal orientations.
2. The AA′ stacking mode exhibits the best mechanical properties among the five different stacking modes. Additionally, the Young’s modulus, the ultimate tensile stress, and the ultimate tensile strain all decrease with an increase in temperature.
3. The natural frequencies of bilayer h-BN nanosheets are influenced by the quantum size effect. As the constraints on the four sides become weaker, the frequency difference between AA′ and AA stacking modes becomes more significant. Specifically, in the AA′ stacking mode, the natural frequencies are the highest, while in the AA stacking mode, they are the lowest. Utilizing nonlocal theory, we propose the free vibration governing equation for four-side simply supported double-orthotropic nanoplate. The theoretical results align with molecular dynamics simulation.

This work provides a fundamental understanding of the mechanical properties and natural frequency of bilayer h-BN nanosheets in different stacking modes, which may shed light on its knowledge in advanced study about h-BN and it is also hoped that this work could be helpful for the applications of bilayer h-BN nanosheets in nanoelectromechanical system.

## Data Availability

The datasets generated and/or analyzed during the current study are available from the corresponding author on reasonable request.
